# The Oxente Chagas Bahia Project: evaluating the efficacy of a rapid diagnostic test and treatments for Chagas disease

**DOI:** 10.1590/0074-02760240140

**Published:** 2024-10-28

**Authors:** Fred Luciano Neves Santos, Tycha Bianca Sabaini Pavan, Cristiane Siqueira Valle, Daniel Dias Sampaio, Larissa Carvalho Medrado Vasconcelos, Maria Hermoso Cristóbal, Ângelo Antônio Oliveira Silva, Cátia Martins de Oliveira, Raquel Santos de Souza, Carmen Nila Phang Romero Casas, André Daher, Isadora Cristina de Siqueira

**Affiliations:** 1 Fundação Oswaldo Cruz-Fiocruz, Instituto Gonçalo Moniz, Laboratório Avançado de Saúde Pública, Salvador, BA, Brasil; 2 Fundação Oswaldo Cruz-Fiocruz, Instituto Gonçalo Moniz, Grupo de Pesquisa Interdisciplinar em Biotecnologia e Epidemiologia de Doenças Infecciosas, Salvador, BA, Brasil; 3 Fundação Oswaldo Cruz-Fiocruz, Vice-Presidência de Pesquisa e Coleções Biológicas, Programa de Pesquisa Translacional em Doenças de Chagas, Rio de Janeiro, RJ, Brasil; 4 Fundação Oswaldo Cruz-Fiocruz, Vice-Presidência de Pesquisa e Coleções Biológicas, Plataforma de Pesquisa Clínica, Rio de Janeiro, RJ, Brasil; 5 Fundação Oswaldo Cruz-Fiocruz, Centro de Desenvolvimento Tecnológico em Saúde, Rio de Janeiro, RJ, Brasil; 6 Fundação Oswaldo Cruz-Fiocruz, Instituto Gonçalo Moniz, Laboratório de Investigação em Saúde Global e Doenças Negligenciadas, Salvador, BA, Brasil

**Keywords:** Chagas disease, rapid diagnostic test, diagnostic performance, cost-effectiveness, antiparasitic treatment

## Abstract

Chagas disease (CD), caused by *Trypanosoma cruzi*, is a life-threatening neglected anthropozoonosis primarily transmitted by triatomine bugs. Affecting an estimated 5.7 million people globally, CD has significant morbidity and mortality, particularly in Latin America. The Oxente Chagas Bahia Project aims to screen approximately 30,000 individuals, validate a rapid diagnostic test in a real-world setting, and provide crucial data on its diagnostic performance and cost-effectiveness. Additionally, a biobank will be established to support further research on disease biomarkers and treatment cure rates. By enhancing access to timely diagnosis and treatment, the project will evaluate a strategy to reduce the CD burden.

Chagas disease (CD) is a life-threatening neglected anthropozoonosis caused by the hemoflagellated kinetoplastid protozoan, *Trypanosoma cruzi*. The parasite is primarily transmitted by blood-sucking insects known as kissing bugs, but can also be spread through contaminated food or beverages (food-borne), vertical transmission (congenital), contaminated blood transfusion, organ transplantation, and laboratory accidents. Globally, an estimated 5.7 million people are infected with *T. cruzi*, resulting in approximately 7,500 annual deaths, predominantly in 21 Latin American countries,^1^ with increasing incidence worldwide due to migration and human mobility.

CD progresses through two phases. During the acute phase, approximately 10% of infected individuals exhibit symptoms such as fever, swelling at the inoculation site, and potentially life-threatening cardiac and nervous system dysfunction. Acute CD diagnosis involves parasitological investigations. Without timely antiparasitic treatment, individuals progress to the chronic phase within weeks, where up to 30% develop cardiac disorders and 10% have gastrointestinal manifestations. At this stage, serological diagnosis using two different methods is recommended.^2^ Early diagnosis and timely antiparasitic treatment are crucial to prevent progression to the chronic phase, that has a high morbidity and requires complex clinical management at high financial and societal costs.

In the chronic phase, antiparasitic treatment is recommended as it lower the frequency of progression to late clinical outcomes.^3^ Additionally, it decreases vectorial and vertical transmission risks by reducing parasite load. In Brazil, benznidazole (BNZ) is the first line treatment. While BNZ is usually well-tolerated in children, adults experience a high rate of adverse events impairing treatment adherence. Nifurtimox (NTF) is an alternative treatment for BNZ-intolerant patients, albeit with similar tolerability concerns. NFT is the rescue treatment to BNZ failures, although cross-resistance is of growing concern.^4^ In Brazil, both medications are not available for purchase and provided free of charge by the National Health System (SUS).

CD is characterised by high morbidity and mortality, significantly impairing quality of life and accounting for over 800,000 disability-adjusted life years (DALYs). The disease imposes a substantial economic burden, costing over $600 million annually in healthcare and resulting in losses exceeding $7 billion to the economy.^5^ ^,^ ^6^ In the early 1990s, the World Bank identified CD as the most hazardous disease in Latin America, underscoring the urgent need for enhanced engagement from healthcare systems. Despite these deleterious impacts, only 7% of individuals infected with *T. cruzi* are aware of their serological status, and merely 1% of these individuals receive etiological treatment.^7^

Several factors contribute to the low rates of diagnosis and treatment for CD. There is a lack of awareness and education among healthcare providers, leading to low levels of screening and diagnosis, particularly in non-endemic areas. Resource limitations in many endemic regions result in inadequate healthcare infrastructure and resources, including insufficient diagnostic tools and medications. Geographic barriers in rural and remote areas of Latin America limit access to healthcare facilities, with public transport being limited or non-affordable. Economic constraints make healthcare costs prohibitive for many people living in poverty, common in CD-endemic regions, limiting their ability to seek diagnosis and treatment. The stigma associated with CD might discourage individuals from seeking medical attention, and the fear of testing positive may prevent people from getting tested. Worldwide there are limited, and uneven availability of treatment caused by the small number of manufactures, and the regulatory and logistical challenges in deploying these medications. The length and safety profile of the treatment regimens deters a high number of patients from adhering to the therapy. Additionally, healthcare systems in some countries are overwhelmed by other public health issues, leaving CD with less attention and resources.

Deployment of new and better diagnosing tools are urgently needed, as the current reference tests for diagnosing chronic CD rely on the combination of two positive serological test results to confirm *T. cruzi* infection.^8^ These laboratory-based tests require adequate infrastructure and skilled operators. Thus, in recent years, international efforts have focused on evaluating the performance of several point-of-care (POC) diagnostic devices, such as rapid diagnostic tests (RDTs).^9^ ^,^ ^10^ ^,^ ^11^ ^,^ ^12^ The widespread use of RTDs as first-line screening tools in the diagnostic algorithm is highly expected to circumvent the need for specialised infrastructure and personnel and increase diagnosis access.

RDTs are designed to be simple, easy, convenient, and intuitive to perform. They do not require refrigeration, specialised infrastructure, trained personnel, or further processing by the user to obtain a result. These devices involve fewer technical procedures, require small sample volumes obtained through capillary or digital puncture, and have a short processing time, providing rapid results in a few minutes. This makes them particularly suitable for rural, remote, and resource-limited settings where traditional laboratory-based methods may not be feasible. RDTs can be performed at the primary care level, close to the community, thus shortening time to treat. A negative RDT result excludes the disease, while positive results should be forwarded for diagnostic confirmation with other serological tests to exclude or confirm CD.^2^ ^,^ ^8^ Recently, the Pan American Health Organization (PAHO) recommended the use of ELISA or RDT as the sole test for seroepidemiological testing.^8^

In line with this strategy, the Institute of Technology on Immunobiologicals (Bio-Manguinhos, Oswaldo Cruz Foundation, Brazil) developed a rapid diagnostic test for CD, the TR Chagas Bio-Manguinhos (FIOCRUZ, Rio de Janeiro, Brazil). This test received commercial production approval from the Brazilian Health Regulatory Agency (ANVISA) in 2020. Initial evaluations using a low number of samples indicated high sensitivity (100%) and specificity (100%).^9^ Subsequent multicentre studies confirmed that TR Chagas Bio-Manguinhos had superior sensitivity and diagnostic odds ratios compared to other commercially available tests, indicating its potential as a screening tool for CD.^12^ Similar results were found in independent studies conducted in Colombia^10^ and Bolivia.^11^ All these studies demonstrated high sensitivity (97.69%-100%) with a moderate specificity (70.85%-78.57%). All these studies were conducted in laboratory environments under controlled conditions, lacking evaluation in real-world scenarios.

The Oxente Chagas Bahia Project (ReBEC approval number RBR-5zfkxdy) aims to implement and validate the TR Chagas Bio-Manguinhos test in a real-world setting, assessing its acceptability and cost-effectiveness for use in the Brazilian SUS. The project is designed as a strategy to achieve two primary outcomes (Figure): (A) enhancing access and demand for CD screening, diagnosis, treatment, and care; and (B) increasing the access to effective etiological treatment as recommended by the Brazilian Ministry of Health’s Clinical Protocol of Therapeutic Guidelines (PCDT) for CD,^2^ tailored to local Discrete Typing Units (DTUs).

**Figure:**
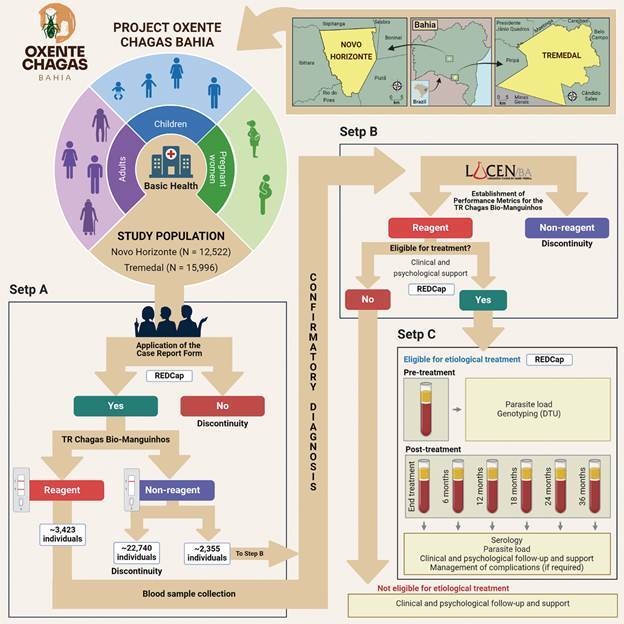
Flowchart illustrating the study design. The study is divided into three steps: (A) seroepidemiological survey using TR Chagas Bio-Manguinhos of residents in Tremedal and Novo Horizonte, Bahia; (B) evaluation of the diagnostic performance and cost-effectiveness of the TR Chagas Bio-Manguinhos for serological screening in a real-world setting; and (C) prospective cohort study assessing the safety and efficacy of Chagas disease treatments.

Led by the Gonçalo Moniz Institute (Fiocruz, Bahia), this project is a collaborative initiative involving key public health stakeholders from the municipalities of Novo Horizonte and Tremedal, in Bahia, Brazil. It is endorsed by the Bahia State Health Department (SESAB), the Institute of Technology on Immunobiologicals (Bio-Manguinhos, Fiocruz) and the Fiocruz Clinical Research Platform. These municipalities were selected due to the residual foci of *Triatoma infestans* observed until 2015 and 2011, respectively. *T. infestans*, the primary vector of *T. cruzi* in endemic areas with domiciliary habits, was eliminated from Brazil in 2006. Although *T. infestans* has not been detected in recent years, other *T. cruzi*-infected triatomine species have been captured both inside and outside houses in these municipalities, thereby providing the conditions to perpetuate the transmission cycle. The impact of these insects on the actual vector transmission of *T. cruzi* remains unknown. Despite the risk of vector transmission, CD has a silent presence in Novo Horizonte and Tremedal, as acute cases compulsory notifications have not been identified in the surveillance system recently. Thus, these study areas are one of the most suitable real-world settings for the validation of TR Chagas Bio-Manguinhos.

Novo Horizonte (12º 48’ 28” S 42º 10’ 04” W) is in the Central-East macro-region of Bahia. The city covers approximately 627 km², has a population of 12,522, and a population density of 17.79 inhabitants/km². The area is predominantly rural, with an economy centred on rutile quartz extraction and garlic cultivation. The municipality is served by two basic health units (UBS) in the urban area and five in rural areas. Tremedal (14º 58’ 21” S 41º 24’ 52” W), another endemic area for CD, is in the Vitória da Conquista micro-region within Bahia’s Central-South macro-region. Tremedal spans 2,010,316 km², has a population of 15,996, and a population density of 8.11 inhabitants/km². It is also predominantly rural, with an economy focused on agriculture. The region experiences a semi-arid climate and has typical backlands vegetation. Tremedal is served by three UBS in the urban area and seven in rural areas.

Through an implementation approach, the Oxente Chagas Bahia Project is divided into three steps (Figure). Step A involves a seroepidemiological survey of residents in Tremedal and Novo Horizonte, Bahia. Step B evaluates the diagnostic performance and cost-effectiveness of the TR Chagas Bio-Manguinhos for serological screening, while Step C comprises a prospective cohort study assessing the safety and efficacy of CD treatments. These components will be conducted concurrently.

Before beginning Step A, a formative education process will be conducted involving all healthcare professionals at the primary care level. A REDCap electronic survey^13^ will be sent for all professionals to assess their knowledge of CD and evaluate the psychosocial and systemic barriers to the test and treat approach. The questions and depth of the topic will be tailored according to the staff roles. For community health agents, the questions will focus on the epidemiology of CD, while for doctors and nurses, in addition to epidemiology, the questions will cover clinical aspects, diagnosis, treatment, and disease monitoring. This strategy will guide our team in identifying the knowledge gaps that need to be addressed during training and follow-up. The subsequent training will include theoretical sessions on key concepts of CD, including CD surveillance, diagnosis, counselling, clinical management, treatment, and the use of the TR Chagas Bio-Manguinhos, as well as study protocol. In addition to in-person training, a series of videos will be produced to train staff that may join the local healthcare teams later on.

The study flowchart is presented in the Figure. All individuals residing in Novo Horizonte and Tremedal, aged over nine months, and registered in the local Citizen’s Electronic Health Record (PEC), will be invited to attend health units for CD testing. Residents willing to participate and that signed the informed consent will be screened using TR Chagas Bio-Manguinhos. If the RDT test result is positive, blood samples will be collected for confirmatory laboratory test at the Central Laboratory of Public Health of Bahia (LACEN-BA), according to national and international guidelines.^2^ ^,^ ^8^ Conversely, if the RDT result is negative, the participant will be discontinued from the study. This study step will provide epidemiological measurements, as the prevalence of CD in the general population, as well as in specific populations such as women of childbearing age, pregnant women, children, the elderly, and immunosuppressed individuals. Furthermore, risk factors will be identified, and risk areas mapped. This information will add great value to design of further local health policies and interventions. The determination of seroprevalence and risk factors will be conducted only after confirmatory testing.

Considering a mean prevalence of approximately 12% of CD in three cities in Bahia,^14^ ^,^ ^15^ it is expected that approximately 3,423 individuals will be identified as positive during screening. These individuals, along with a percentage (~ 10%) of negative individuals, will have blood collected for confirmatory diagnosis at LACEN-BA. This quantitative sample will be used to determine the performance parameters of the TR Chagas Bio-Manguinhos in a real-world setting. Metrics such as sensitivity, specificity, accuracy, positive and negative predictive values, positive and negative likelihood ratios, Cohens’ *Kappa*, and diagnostic odds ratio will be calculated along with their 95% confidence intervals (95%CI). These activities comprise Step B, with the main outcomes including the implementation and acceptability of the TR Chagas Bio-Manguinhos at the primary care level of two CD endemic cities in Bahia, and the scientific evidence supporting its use as screening test.

An economic evaluation will compare TR Chagas Bio-Manguinhos and diagnostic test with the standard diagnostic tests performed by LACEN- BA among individuals attending primary care centres. A micro-costing study using an ingredients-based approach will be conducted to estimate the total resources used for screening in the participating municipalities. This data will support Cost-Effectiveness Analyses and assess the Budget Impact (BI) on the Brazilian Health Care System. A decision tree model will be constructed to compare the TR Chagas Bio-Manguinhos test with the reference tests performed by LACEN-BA. This model will integrate biological, epidemiological, and economic data, among other sources, to estimate the cost-effectiveness of each strategy. For the BI analysis, the healthcare provider perspective will be used to estimate the incremental annual financial expenditure required for the potential introduction of the new rapid test in the SUS.

Step C will be performed exclusively with individuals confirmed to be positive for CD and eligible for etiological treatment against *T. cruzi*, according to criteria established by the PCDT.^2^ Participants in Step C will undergo comprehensive clinical and laboratory assessments, including electrocardiogram (ECG), X-ray, and various laboratory tests (*e.g.*, blood count, renal function, liver function, C-reactive protein, uric acid, total protein). For women of childbearing age or those suspected of pregnancy, a pregnancy test (β-hCG) will also be conducted. Blood samples will be collected from all participants before treatment to assess parasite load using NAT Chagas and to genotype the parasite. During treatment, participants will undergo clinical examination and laboratory evaluations to monitor for side effects. Follow-up clinical and psychological evaluations will be conducted at several time points post- treatment initiation: immediately after treatment completion, and at six, 12, 18, 25, and 36 months. At each follow-up, blood samples will be collected to evaluate antibody titres and parasite load using NAT Chagas. Through Step C, we aim to treat all eligible *T. cruzi-*positive individuals, improving their quality of life and reducing the risk of disease progression to symptomatic forms.

The protocol for pregnant women also includes comprehensive screening, diagnosis, follow-up, and treatment measures. Initial screening will be performed using the TR Chagas Bio-Manguinhos RDT, with positive results confirmed by standard laboratory methods at LACEN-BA. Pregnant women who test positive for CD will receive enhanced follow-up and monitoring to reduce the risk of vertical transmission, including post-delivery molecular testing of the newborn. Treatment during pregnancy is deferred due to teratogenic risks; however, both the mother and newborn, if confirmed positive by NAT Chagas, will receive BNZ therapy after delivery, following the PCDT for CD.

In summary, the Oxente Chagas Bahia Project is designed to address key challenges in CD management by validating the TR Chagas Bio-Manguinhos RDT in real-world settings, assessing its cost-effectiveness for routine screening, and incorporating molecular tests to evaluate treatment efficacy. While the TR Chagas Bio-Manguinhos RDT has shown high sensitivity and specificity in controlled environments, its performance under typical conditions where factors such as sample quality, operator expertise, and environmental variables come into play remains unverified. This project aims to validate the RDT in two endemic cities of Bahia by evaluating its diagnostic accuracy, feasibility, and acceptability in primary care settings. The outcomes of this study will inform its broader implementation within SUS. Validation in real-world conditions is crucial as it may reveal variability not observed in controlled studies, such as differences in patient demographics, environmental influences, and operator proficiency. Additionally, the moderate specificity observed in previous studies raises the risk of false positives, necessitating confirmatory testing, which could limit the standalone utility of the RDT in certain settings. The project’s success is also contingent on the local infrastructure and the capacity of health units, which could affect the scalability of the findings to regions with different healthcare capabilities.

Incorporating RDTs into routine screening programs has the potential to reduce costs, enhance access to early diagnosis, and improve the overall efficiency of CD management. To support this, a micro-costing study will estimate the resources required for screening, while a decision tree model will compare the TR Chagas Bio-Manguinhos RDT to standard diagnostic methods. This economic evaluation is intended to guide policymakers on the cost-effectiveness of integrating the RDT into the SUS, ultimately supporting its broader adoption and resource allocation. However, this analysis relies on assumptions about disease prevalence, test accuracy, and healthcare costs that may not be applicable across all regions or populations. Local variations in healthcare delivery, patient adherence, and follow-up costs could influence the economic model’s projections, complicating the generalisation of the findings. Moreover, the study’s dependence on existing healthcare infrastructure may not fully account for the challenges faced in more remote or resource-limited settings, potentially affecting the applicability of its conclusions.

The inclusion of molecular tests such as NAT Chagas is intended to enhance treatment monitoring and outcome assessments. These tests provide precise measurements of parasite load, offering critical tools for evaluating the efficacy of CD treatment regimens. By integrating molecular tests, the project seeks to gain deeper insights into treatment outcomes, particularly in confirming treatment failure and monitoring disease progression post-treatment. However, the complexity, cost, and infrastructure requirements of molecular tests may limit their scalability in resource-constrained settings. In this project, the NAT-Chagas test will specifically be used to identifying therapeutic failure rather than for broader integration into public health systems or for economic evaluation. All molecular analysis will be performed in a controlled-environment laboratory at Fiocruz, Bahia.

Additionally, all biological samples collected during the study will be stored in a biobank and biorepository at Fiocruz-BA for future studies on disease progression biomarkers and etiological cure. All participants study data including the informed consent will be collected using REDCap’s Case Report Forms (CRF). The CRFs includes socio-demographic descriptors, evaluations of CD, triatomines previous contact, and participants’ health data. The final data set will be made available to the scientific community upon request in ARCADados, the Fiocruz data repository.

At the completion of the Oxente Chagas Bahia Project, it is anticipated that approximately 30,000 individuals will be screened, with those confirmed as positive for CD will directly benefiting from the test-treat-care approach. This initiative aims to validate the TR Chagas Bio-Manguinhos test in a real-world setting, providing critical data on its diagnostic performance and cost-effectiveness to support its broader implementation in the SUS. By increasing access to accurate and timely diagnosis, the project will facilitate the prompt initiation of antiparasitic treatment, thereby reducing the burden of CD in the study area. Furthermore, the establishment of a biobank for future research on disease progression biomarkers and etiological cure will further enhance our understanding and management of this neglected tropical disease. Overall, the Oxente Chagas Bahia Project exemplifies a comprehensive and collaborative approach to addressing the multifaceted challenges of CD, paving the way for more effective CD public health strategies and interventions.

*Ethical approval statement* - This study received approval from the Institutional Review Board for Human Research at the Gonçalo Moniz Institute (IRB/IGM/Fiocruz-BA), Salvador-Bahia, Brazil, with protocol number 70324323.0.0000.0040. All participants will provide written informed consent, and strict measures will be taken to ensure the privacy of patient data.

## References

[1] WHO (2024). Chagas disease (also known as American trypanosomiasis). https://www.who.int/news-room/fact-sheets/detail/chagas-disease-(american-trypanosomiasis).

[2] MS (2018). Protocolo clínico e diretrizes terapêuticas doença de Chagas. : Ministério da Saúde/Secretaria de Ciência, Tecnologia e Insumos Estratégicos.

[3] Pérez-Molina JA, Pérez-Ayala A, Moreno S, Fernández-González MC, Zamora J, López-Velez R (2009). Use of benznidazole to treat chronic Chagas' disease a systematic review with a meta-analysis. J Antimicrob Chemother.

[4] Dias JCP, Ramos AN, Gontijo ED, Luquetti A, Shikanai-Yasuda MA, Coura JR (2016). II Brazilian Consensus on Chagas Disease, 2015. Rev Soc Bras Med Trop.

[5] Martins-Melo FR, Carneiro M, Ramos AN, Heukelbach J, Ribeiro ALP, Werneck GL (2018). The burden of neglected tropical diseases in Brazil, 1990-2016: a subnational analysis from the Global Burden of Disease Study 2016. PLoS Negl Trop Dis.

[6] Lee BY, Bacon KM, Bottazzi ME, Hotez PJ (2013). Global economic burden of Chagas disease a computational simulation model. Lancet Infect Dis.

[7] Chaves GC, Arrieche MAS, Rode J, Mechali D, Reis PO, Alves RV (2017). Estimación de la demanda de medicamentos antichagásicos una contribución para el acceso en América Latina. Rev Panam Salud Publica.

[8] PAHO - Pan American Health Organization (2019). Guidelines for the diagnosis and treatment of Chagas disease.

[9] Silva ED, Silva AAO, Santos EF, Leony LM, Freitas NEM, Daltro RT (2020). Development of a new lateral flow assay based on IBMP-8 1 and IBMP-8.4 chimeric antigens to diagnose Chagas disease. Biomed Res Int.

[10] Marchiol A, Sanchez ACF, Caicedo A, Segura M, Bautista J, Sotelo MSA (2023). Laboratory evaluation of eleven rapid diagnostic tests for serological diagnosis of Chagas disease in Colombia. PLoS Negl Trop Dis.

[11] López R, García A, Aruni JJC, Balboa V, Rodríguez A, Erkosar B (2024). Comparative evaluation of lateral flow assays to diagnose chronic Trypanosoma cruzi infection in Bolivia. PLoS Negl Trop Dis.

[12] Iturra JAD, Leony LM, Medeiros FAC, Souza JA, Siriano LR, Tavares SB (2023). A multicenter comparative study of the performance of four rapid immunochromatographic tests for the detection of anti-Trypanosoma cruzi antibodies in Brazil. Front Med (Lausanne).

[13] Harris PA, Taylor R, Minor BL, Elliott V, Fernandez M, O'Neal L (2019). The REDCap consortium building an international community of software platform partners. J Biomed Inform.

[14] Martins-Melo FR, Ramos AN, Alencar CH, Heukelbach J (2014). Prevalence of Chagas disease in Brazil a systematic review and meta-analysis. Acta Trop.

[15] Pavan TBS, Dias DP, Cangussú MM, Dutra VPP, Sampaio DD, Santos FLN (2023). Seroepidemiology of Chagas disease in at-risk individuals in Caraíbas, a city with high endemicity in Bahia State, Brazil. Front Public Health.

